# Different Role of *Caveolin-1 *Gene in the Progression of Gynecological Tumors

**DOI:** 10.31557/APJCP.2019.20.11.3259

**Published:** 2019

**Authors:** Yan Gong, Yuhan Yang, Sufang Tian, Honglei Chen

**Affiliations:** 1 *Department of Pathology, Zhongnan Hospital of Wuhan University, *; 2 *Department of Pathology, School of Basic Medical Science, Wuhan University, Wuhan, P. R. China. *

**Keywords:** Caveolin-1, cancer progression, gynecological tumor

## Abstract

*Caveolin-1 (Cav-1)*, an integral membrane protein, is a principal component of caveolae and has been reported to play a promoting or inhibiting role in cancer progression. Gynecologic tumor is a group of tumors that affect the tissue and organs of the female reproductive system, especially cervical cancer. Cervical cancer, as one of the most common cancers, severely affects female health in developing countries in particular because of its high morbidity and mortality. This review summarizes some mechanisms of* Cav-1* in the development and progression of gynecological tumors. The role of *Cav-1* in tumorigenesis, including dysregulation of cell cycle, apoptosis and autophagy, adhesion, invasion, and metastasis, such as the formation of invadopodia and matrix metalloproteinase degradation are presented in detail. In addition, *Cav-1* modulates autophagy and the formation of invadopodia and target regulated by miRNAs to affect tumor progress. Taken together, we find that, no matter *Cav-1 *expression in the tumor or stromal cells , *Cav-1 *has paradoxical role in different types of gynecological tumors in vivo or in vitro and even in the same tumor from the same organ.

## Introduction

Since the identification of caveolae in epithelial cells of capillary and gall bladder of the mouse in 1950’s, it has been found to exist in a wide variety of cell types and tissues, especially in terminally differentiated cells, such as adipocytes, endothelial cells, epithelial cells, fibroblasts, and smooth muscle cells (Scherer et al., 1997). Except for many studies on its location, some progress has also been made regarding the function of caveolae. Caveolae plays an important role in macromolecular transport vesicles, signal transduction, endocytosis, cellular metabolism, cholesterol homeostasis, and tumor progression (Razani et al., 2000b; Galbiati et al., 2001a; Matveev et al., 2001; Parton and Richards, 2003).

Caveolins is an integral membrane protein, whose family includes three members, including caveolin-1/*Cav-1*, caveolin-2/Cav-2 and caveolin-3/Cav-3), is a principal component of caveolae plasma membranes (Glenney and Soppet, 1992). *Cav-1*, the mostly documented one, has been reported to be down-regulated in different types of human tumor samples, suggesting negative correlation between its expression level and tumor formation and progression, so acting as an tumor suppressor (Bagnoli et al., 2000; Bender et al., 2000). However, *Cav-1* has been discovered to be up-regulated in the advanced tumor stage of different kinds of cancers, demonstrating its association with invasion and metastasis (Ho et al., 2002). The different roles of *Cav-1* in cancer may depend on the stage of cancer and its interaction with multiple different signaling molecules in specific tissues and cell types, thus it remains controversial whether *Cav-1* acts as an tumor-promoter or a suppressor (Fu et al., 2017).

In order to have a better understanding of the apparent ‘‘*Cav-1* paradox”, a “*Cav-1* + partner-X” model has been proposed which considers “*Cav-1* + partner-X” as a function integration, but not *Cav-1* alone (Burgermeister et al., 2008). For example, *Cav-1* displays its role as a tumor-suppressor requiring the presence of E-cadherin (Torres et al., 2007). When E-cadherin is still expressed in cancer cells, *Cav-1* keeps its anti-proliferative and pro-apoptotic role; whereas, the depletion of E-cadherin resulting in loss of *Cav-1* function. Caveolae has been regarded as a message centers, and many signaling molecules taking part in different signaling pathways focus on caveolins areas of the cell surface (Anderson, 1993). Through direct interaction with different signaling molecules by its scaffold areas or regulation of the internalization of signaling molecules to reduce the signals (Burgermeister et al., 2008), the complex of caveolins either promote or suppress tumor occurrence. According to its expression level, *Cav-1* is mainly regarded as a tumor suppressor in the early stage of carcinoma since its expression is generally down-regulated in transformed cells; but *Cav-1* is suggested to promote cancer progression for its up-regulation in the later stage of some advanced carcinomas (Nunez-Wehinger et al., 2014).

Gynecologic tumors are a group of tumors that affect the tissue and organs of the female reproductive system, which often involve the uterus, ovaries, cervix, vulva, vagina, fallopian tubes, or the peritoneum. The most common gynecologic cancer in China is cervical cancer, followed by ovarian cancer and endometrial cancer (Torre et al., 2015). Gestational trophoblastic disease is a gynecologic tumor that may behave aggressively whether being malignant or not. Having a better understanding of the mechanism in tumor progression will greatly contribute to clinical diagnosis, targeted therapy, and prognostic evaluation. Although the role of *Cav-1* has been reported in many kinds of tumors, there are a few reports about the role of *Cav-1* in gynecological tumors. Therefore, this review summarized some mechanisms and functions of *Cav-1* in the development and progression of gynecological tumors.

*Cav-1 Fuction in Carcinogensis, Invasion, and Metastasis*

Tumor formation is a process of interaction among multiple molecules during a long time, dividing into different stages and presenting some specific characteristic in each stage. In the early stage, the principal feature is abnormal proliferation of tumor cells, while invasion and metastasis are remarkable at the later stages. The role of *Cav-1* in tumorigenesis, including dysregulation of cell cycle, apoptosis and autophagy, adhesion, and invasion and metastasis, such as the formation of invadopodia and matrix metalloproteinase degradation is presented in detail in the following sections.

*Cav-1 inhibits cell cycle*

**Figure. 1:**
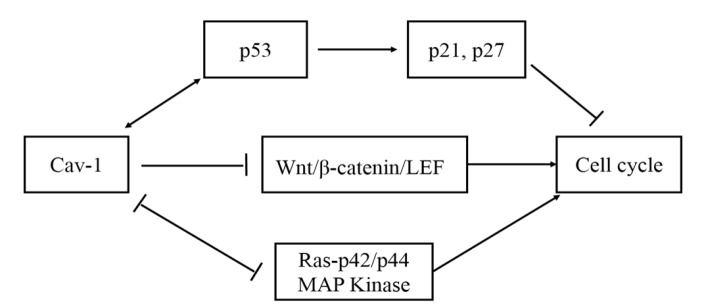
The Mechanism of *Cav-1* Inhabits Cell Cycle. There are three pathways: enhancing the activity of p53 to promote downstream expression of p21 and p27; inhibiting Wnt/β-catenin/LEF pathway; negatively regulating Ras-p42/p44 MAP kinase pathway

*Cav-1* has been reported to interact with signaling molecules to regulate signaling pathways, such as Wnt, Ras, and p53 and have great effect on inhibiting cell mitogenic and modulating cell cycle. p53 is a key factor in cell cycle arrest and apoptosis promotion through inducing the expression of downstream p21 and p27. Previous studies showed that *Cav-1* could mediate cell cycle arrest through p53/p21 pathway, inducing cells to stay at the G0/G1 stage, reducing cell proliferation and DNA replication rate, because of the increased activity of p53 in NIH 3T3 cells transiently expressing *Cav-1* (Galbiati et al., 2001b). P53 can positively regulate the transcription of *Cav-1* gene and protein expression (Razani et al., 2000a). Moreover, the expression of *Cav-1* can enhance the activity of p53Cav-1 can also negatively regulate Wnt/β-catenin/LEF pathway, that is another kind of mitogenic pathway. Cav-1 arrests β-catenin in the caveolin-rich membrane domains and stables the cell adhesion, thus inhibiting β-catenin to enter nuclear; in addition, it interacts with LEF1 to initial transcription (Galbiati et al., 2000). In agreement, *Cav-1* inhibits maximally the cyclinD1 gene promoter, depending on the T-cell factor/lymphoid enhancer factor-1 (TCF/LEF-1) binding site between -81 to -73 in promoter area, which occurs mutation and is abolished the ability to bind TCF protein (Hulit et al., 2000). Furthermore, *Cav-1* can inhibit cell cycle through modulating the Ras-p42/p44 MAP kinase pathway. In precise, there is a reciprocal negative regulation relationship between the activation of p42/p44 MAP kinase and *Cav-1* expression. In other words, increasing *Cav-1* expression can down-regulate p42/p44 MAP kinase activity; on the contrary, enhancing p42/p44 MAP kinase activity can decrease *Cav-1* expression (Engelman et al., 1998). These data demonstrate that *Cav-1* can be considered as a tumor-suppressor which is able to limit cell proliferation in the early stage of tumor progression (Figure 1).

*Cav-1 promotes cell apoptosis and modulates autophagy*

The PI-3 kinase pathway plays an important role in stimulating cell proliferation, anti-apoptosis, and pro-survival. In the circumstances of desensitization of pro-apoptosis activity resulted from the transfection of *Cav-1* antisense vector, the pro-apoptosis activity can restore sensitivity through blocking PI-3 kinase -mediated survival signaling (Liu et al., 2001). Ceramide is reported to have the ability to inhibit PI-3 kinase activity through recruiting PI-3 kinase to *Cav-1*-enriched membrane domains (Zundel et al., 2000), thus inducing cell apoptosis. Up-regulation of *Cav-1* is adequate to limit PI-3 kinase activity and attributes to ceramide-induced apoptosis ; whereas, down-regulation of *Cav-1* by antisense repression inversely activates PI-3 kinase and reduces ceramide-induced cell apoptosis. In precise, *Cav-1* interacts with epidermal growth factor receptor (EGFR) and p85, the regulatory subunit of PI-3 kinase, to form a complex, inhibiting PI-3 kinase signaling pathway by inactivating PI-3 kinase, thus enabling ceramide-induced apoptosis. Since EGFR is a component of the complex, the sensitivity of apoptosis may be linked to the inhibition of cell proliferation, because the overexpression of *Cav-1* can inhibit the EGF-induced p42/p44 MAP kinase signaling pathway(Engelman et al., 1998), consistent with the report that *Cav-1* expression can promote apoptosis activity mediated by inhibition of EGF-induced survival pathways (Razani et al., 2001). Another study showed that *Cav-1* plays a role as sensitizing factor or a component of sensitizing complex for cell apoptosis in two cells, namely epithelial (T24) cell and fibroblastic (NIH/3T3) cell (Liu et al., 2001). The above results identified that the character of *Cav-1* to inducing pro-apoptosis in agreement with its role acting as an anti-oncogene in the early stage of tumor progression .

**Figure 2:**
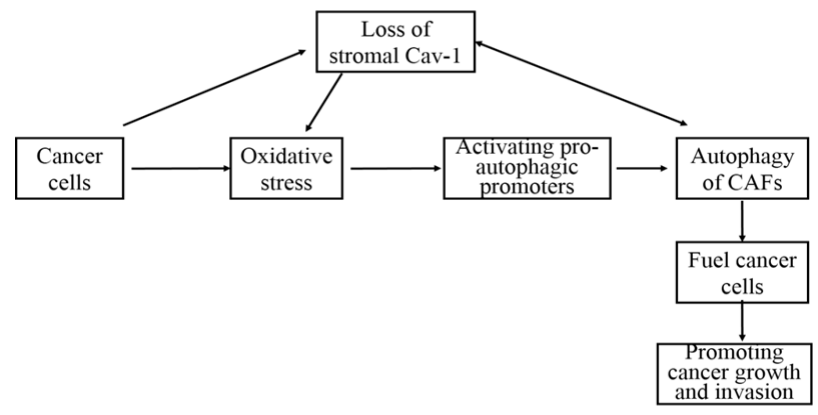
The Association between Stromal *Cav-1*, Oxidative Stress and Cancer Progression in Tumor Microenvironment

It has been proposed that the absence of stromal *Cav-1* via autophagy is a negative prognostic factor during tumor progression (Martinez-Outschoorn et al., 2010b; Wu et al., 2018; Qian et al., 2019). The co-culture of human breast cancer cells (MCF7) and immortalized fibroblasts (hTERT-BJ1) or normal human mammary fibroblasts leads to autophagic/lysosomal degradation and down regulation of *Cav-1* in fibroblasts, resulting in cancer associated fibroblast (CAFs) phenotype(Martinez-Outschoorn et al., 2010a). To understand tumor metabolism and its invasion through tumor stromal autophagy, a model has been established (Figure 2) (Trimmer et al., 2011). In this model, autophagy in tumor stroma provides fuel for cancer cells, enabling the aggressive growth towards periphery. Mechanically, cancer cells induce the activity of autophagy in CAFs through the loss of *Cav-1*, promoting oxidative stress in adjacent stromal fibroblasts, and subsequently resulting in promoting tumor growth. Cancer cells can also trigger oxidative stress in the microenvironment and activate pro-autophagic promoters in CAFs (Chen and Che, 2014). The autophagy of stromal fibroblasts results in the autophagic loss of *Cav-1*, and the loss of stromal *Cav-1* exacerbates oxidative stress and further promotes autophagy(Zhao et al., 2013). However, contradictory result shows that stromal *Cav-1* has a potential to facilitate tumor invasion and metastasis by regulating Rho activity, p190 localization, and phosphorylation (Goetz et al., 2011). 

Besides, *Cav-1* also modulates autophagy of tumor cells, significantly impacting cancer progression. *Cav-1* deficiency promotes cell autophagy in breast cancer, and its promoting effect mainly appears at the late stage of autophagy by increasing lysosomal function and autophagosome-lysosome fusion. Furthermore, the elevated autophagy level induced by *Cav-1* deficiency can be regarded as a cell survival mechanism under starvation (Shi et al., 2015). Interestingly, *Cav-1* can regulate cell apoptosis and autophagy at the same time through 17β-estradiol-mediating pathway in human breast cancer cell line, BT474 (Wang et al., 2014). The abundant expression of *Cav-1* promotes autophagy and represses cell apoptosis, while deletion of *Cav-1* by siRNA represents the adverse result. Except for breast cancer, expression of *Cav-1* contributes to tumor growth and metastasis through inhibiting autophagy in hepatocellular carcinoma (Liu et al., 2016). In general, the loss of stromal *Cav-1* through autophagy is a negative prognostic factor during tumor progression, while the autophagy of tumor cells modulated by *Cav-1* has different function in cancer progression.

*Cav-1 enhances cell adhesion*

**Figure 3:**
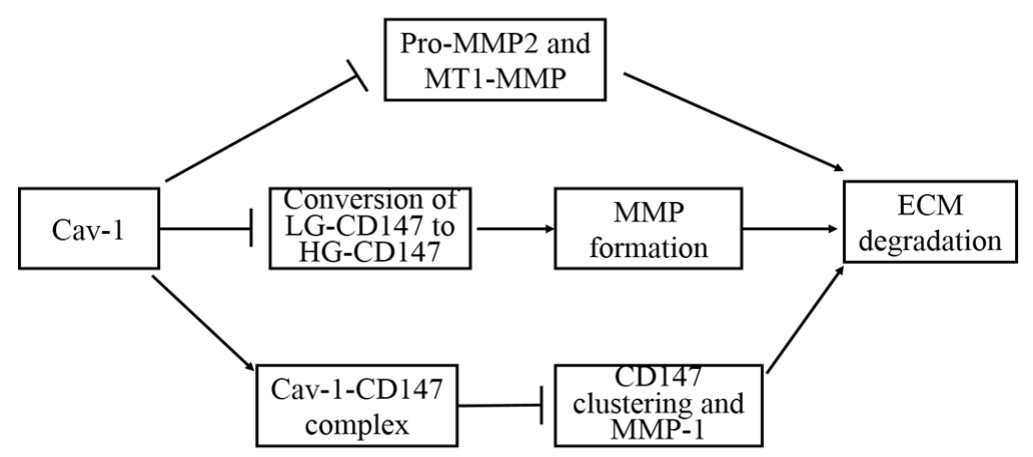
Expression of *Cav-1* can Enhance Cell Adhesion. Up-regulation of *Cav-1* expression can recruit β-catenin to caveolae membranes to stable adhesion complexes, thereby, reinforcing cell-cell adhesion; at the same time, prevent the entrance of β-catenin to cell nucleus and inhibit β-catenin/LEF-1 signal. EGF binding with *EGFR* can down-regulate *Cav-1* and* E-cadherin* expression, inducing β-catenin separating from E-cadherin to nucleus and enhancing β-catenin-TCF/LEF-1 signal

Cadherin is an important adhesion related molecule, which is supposed to be an invasion-suppression factor acting as an anti-oncogene. *Cav-1* has been validated to maintain epithelial and endothelial barriers as a participating protein targeted to adherens and tight junction (Miotti et al., 2005; Schwarz et al., 2007). Thus, the interaction between *Cav-1* and cadherin may greatly influence cell adhesion and tumor cell metastasis (Figure 3). β-catenin is a component of adhesive complexes, which stables adhesion between adjacent cells. However, when it is separated from E-cadherin and translocated to cell nucleus, β-catenin initials gene transcription. Therefore, both expression and distribution of β-catenin are critical in modulating cell-cell adhesion and gene transcription. Up-regulation of *Cav-1* expression has been reported to recruit β-catenin to caveolae membranes to stable adhesion complexes and reinforce cell-cell adhesion; meanwhile, prevent the entrance of β-catenin to cell nucleus and inhibit β-catenin/LEF-1 signaling pathway (Galbiati et al., 2000). EGF-induced down-regulation of *Cav-1* plays a vital role in the complexe changes leading to metastasis (Lu et al., 2003). It is found that chronic EGF treatment can down-regulate *Cav-1* and E-cadherin expressions, inducing β-catenin separation from E-cadherin and translocation to nucleus and enhancing β-catenin-TCF/LEF-1 signaling and transcriptional activity, subsequently resulting in disruption of cell-cell adhesion and epithelial-to-mesenchymal transition (EMT). These data suggest that *Cav-1* expression promotes cell adhesion, thus can suppress the tumor progression.

The main feature of normal cell growth is anchorage-dependent proliferation, it going to anchorage-independent induced apoptosis upon cell detachment. When cell detaches, the signal subsequently triggers *Cav-1* endocytosis accompany by down-regulating integrin signaling and inducing apoptosis (del Pozo et al., 2005; Del Pozo and Schwartz, 2007). Signaling pathways, such as Erk, PI-3 kinase and Rac need integrin-mediated cell adhesion. On the other hand, the components of these signaling pathways locate to lipid rafts, the same place caveolins localization. According to caveolae signaling hypothesis, various inactive signaling molecules are located in caveolar, laying the basis of subsequent molecules activation and cross-talk between different signaling pathways (Lisanti et al., 1994). Integrin-mediated adhesion can modulate lipid rafts trafficking such that upon cell detachment, the phosphorylation of *Cav-1* and its location change from focal adhesions to caveolae induce lipid rafts internalization and clearance, resulting in inhibition of PI-3 kinase, Erk, and Rac, thus suppressing tumor growth (del Pozo et al., 2005). The phenomenon that *Cav-1* promotes cells apoptosis or down-regulates downstream signaling pathways following cells detachment is beneficial for the prevention of tumor formation.

*Cav-1 and invadopodia*

Some documents demonstrate that *Cav-1* represses the formation of invadopodia. A relationship between *Cav-1*, cholesterol and invasion in cancer progression indicates that lower cholesterol is beneficial to inhibit the formation of invadopodia (Caldieri et al., 2009). The appropriate levels of cholesterol in plasma membrane decide the formation, structural integrity, and function of invadopodia. On the other hand, *Cav-1* is a key regulator for invadopodia formation acting through modulating cholesterol levels at the plasma membrane. The phosphorylation of *Cav-1* tyrosine impedes the formation of invadopodia by mediating the internalization of cholesterol enriched membrane microdomains (CEMM) from the plasma membrane upon cell detachment. Similarly, metastatic mammary adenocarcinoma cells (MTLn3) which represents a rapid lamellipod extension and cell migration induced by EGF fails to express endogenous *Cav-1* at detectable levels (Zhang et al., 2000). Re-expression of *Cav-1* in MTLn3 can inhibit the EGF-induced lamellipod biogenesis and cell migration, prevent the anchorage-independent growth, and suppress the invasion and metastasis of tumor cells.

However, contradictory views in this regard represent a promoting role in invadopodia formation. Cell polarization and directional movement can be prevented by *Cav-1* polarity loss because of the targeted down-regulation of *Cav-1* (Beardsley et al., 2005). In the process of cells migration, *Cav-1* is found to be in the posterior of moving cells. In addition, *Cav-1* and lipid rafts are required for the formation and activity of invadopodia and extracellular matrix (ECM) degradation in human breast cancer cells (Yamaguchi et al., 2009). *Cav-1* and membrane type 1 matrix metalloproteinase (MT1-MMP) co-localize and co-express in invadopodia, resulting in ECM degradation and tumor invasion and migration (Ebisawa et al., 2015; Yang et al., 2016). The activation of *Cav-1* and PI3K/Akt/mTOR signaling under low shear stress increases the MT1-MMP expression, invadopodia formation, and ECM degradation and promotes breast cancer cell motility and metastasis(Yang et al., 2016). These results seem to be contradictory regarding the proposed function for *Cav-1*, a tumor suppressor, since the expression of *Cav-1* is a promoting factor in invadopodia formation and migration ability.

*Cav-1 inhibits ECM degradation*

**Figure 4:**
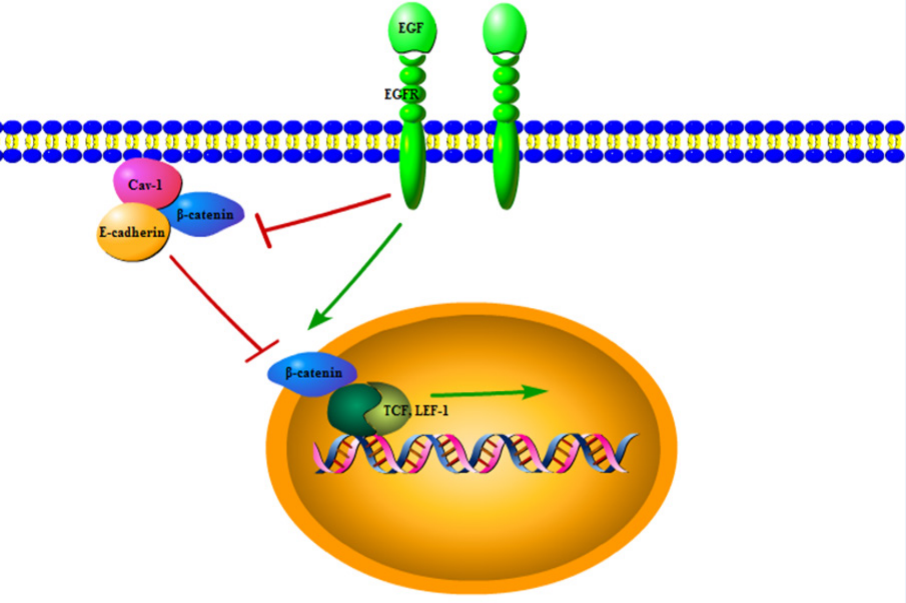
*Cav-1* Inhibits ECM Degradation Through Repressing the Formation and Activity of MMP. Inhibiting the activity of Pro-MMP2 and MT1-MMP through MT1-MMP internalization; restraining the conversion of low glycosylation *CD147 (LG-CD147)* to high glycosylation (HG-CD147) to decrease MMP formation; Forming *Cav-1*/*CD-147* complex to diminish CD147 clustering in the cell surface and CD147-dependent MMP-1 activity

The degradation of ECM contributes to tumor cells invading into blood and lymphatic vessels and promotes tumor invasion and metastasis. *Cav-1* is suggested to attenuate proMMP-2 activity and down-regulate MT1-MMP activity by promoting internalization of MT1-MMP from the cell surface (Kim and Chung, 2008), consequently resulting in ECM cleavage reduction and cell migration inhibition. MT1-MMP can degrade ECM and activate proMMP-2, thus increasing the degradation of ECM, promoting ECM remodeling, and contributing to cancer cell invasion and metastasis. In murine mammary glands, loss of *Cav-1* can increase the expression of ECM and α-SMA protein and change ductal architecture, which may lead to tumor initiation(Thompson et al., 2017). In addition, *Cav-1* has been reported to form a complex with CD147, diminishing CD147 clustering in the cell surface and CD147-dependent MMP-1 activity (Tang and Hemler, 2004). Also, *Cav-1* is demonstrated to restrain the conversion of low glycosylation CD147 to high glycosylation (Tang et al., 2004), inhibiting MMP induction and ECM degradation and acting as tumor suppressor because high glycosylation CD147 can stimulate the induction of MMP (Figure 4). Through modulating MMP activity and formation and reducing MMP-induced ECM degradation, *Cav-1*negatively regulates metastasis progression as an anti-oncogene.

*Cav-1, Cav-2, and miRNAs*

Many documents have demonstrated that caveolins are the target of miRNAs in regulating tumor cells proliferation, survival, invasion, and migration. *MiR-124-3p,* -200c-3p, and -30a-5p are the most influential miRNAs, which are down-regulated in clear cell renal cell carcinoma (ccRCC) through constructing miRNA-target interaction network (Butz et al., 2015). Mechanically, miR-124-3p has a key role in repressing tumor progression by targeting *Cav-1* and Flotillin 1 (FLOT1). The higher expression of *Cav-1* and FLOT1 is associated with lower *miR-124-3p* expression and poor survival of ccRCC. Restoration of the expression of these miRNAs can reduce the proliferation, invasion, and migration of tumor cells. Thus, re-expression of these miRNAs can be considered as a potential therapeutic strategy in ccRCC. These results consist with those of a previous research revealing that *miR-133a* regulates the expression of *Cav-1* which mediates tumor cell invasion and migration in head and neck squamous cell carcinoma (SCC) (Nohata et al., 2011). Here, mi-RNAs inhibit cancer progression, while caveolins promote it; moreover, the expression level of caveolins is negatively regulated by mi-RNAs. Conversely, miR-107 is highly expressed in the pancreatic ductal adenocarcinoma tissues and cells, which is associated with poor prognosis. Low expression of miR-107 due to the inhibitor transfection inhibits cell migration and invasion by up-regulating *Cav-1* and PTEN (Xiong et al., 2017).

MiR-203 is suggested to repress tumor invasion and migration through *Cav-1* in pancreatic cancer cells (Panc-1 cells) (Miao et al., 2014). However, miR-203 positively regulates the expression of *Cav-1* and plays a synergistic effect in inhibiting tumor invasion and migration in Panc-1 cells. Besides, *Cav-1* is essential for miR-203-dependent tumor cells invasion and migration inhabitation. Transfection of miR-203 alone in *Cav-1* knockdown Panc-1 cells fails to suppress cell invasion and migration, but significantly elevates cell migration.

Except for *Cav-1*, Cav-2 is also reported as a target of mi-RNAs in MDA-MB-231 and MT-1 breast cancer cell lines (Shatseva et al., 2011). MiR-199a-3p promotes cell proliferation and survival in MDA-MB-231, MT-1 breast cancer cells, and YPEN-1 endothelial cells lines by inhibiting endogenous and exogenous *Cav-2*. Overexpression of *Cav-2* completely offsets the effect of MiR-199a-3p in cell proliferation and survival. In a previous study, MiR-199a-3p contributed to breast cancer progression; whereas, *Cav-2* repressed cancer progression. In contrast, *Cav-2*, targeting negatively regulated by miR-218 , was reported to promote cell invasion and migration in renal cell carcinoma through dysregulation of focal adhesion (Yamasaki et al., 2013).

Although the promoting or inhibiting role of miRNAs and caveolins in different types of cancer is reported, the targeting role of caveolins regulated by miRNAs in cancer progression has been identified clearly.

*CAV1 Expression in Gynecological Tumor*

The expression of *Cav-1* is distinct in different cell types and tissues and also in different stages of tumor(Fu et al., 2017), so the levels of *Cav-1* mRNA and protein are valuable to be discussed in gynecological tumor. The role of *Cav-1* in gynecological tumor is also paradoxical acting a promoting or inhibiting role in tumor progression (Table 2, Table 3). or not a potential biomarker of uterine malignant mesenchymal tumors (Hayashi et al., 2015). On the other hand, there are very few reports on the role of *Cav-1* expression in vaginal cancer, vulvar cancer, and fallopian tube cancer. In the following, paradoxical roles of *Cav-1* are explained in more details.

*The promoting role of Cav-1*

Some studies have suggested that *Cav-1* promotes tumor progression. In other words, higher expression of *Cav-1* contributes to the incidence and development of gynecological tumor. In cervical SCC, expression of *Cav-1* in tumor cells possibly contributes to cancer progression through enhancing cell proliferation, but little connection was reported between stromal tissues and *Cav-1* expression (Sun et al., 2012). Through gene ontology analysis, *Cav-1* was revealed as one of key node genes for cervical SCC invasion and metastasis (Cheng et al., 2015). In ovarian cancer, it was found that the expression of *Cav-1* was frequently presented in advanced ovarian carcinomas and more expressed at cell membrane with metastasis and poor-survival tumors (Davidson et al., 2001). Besides, it was revealed that *Cav-1* promoted chemotherapy resistance in ovarian cancer through inhibiting cell apoptosis in the Notch-1/Akt/NF-κB pathway (Zou et al., 2015). In a nutshell, it can be concluded that cisplatin-induced apoptotic ratio is higher in common ovarian cancer compared with ovarian cancer cells with cisplatin-resistance; moreover, it seems that down-regulation of *Cav-1* expression by knockdown of *Cav-1* can increase cisplatin-induced cell apoptosis.

Cancer cells metastasis significantly promotes cancer progression. Up-regulated expression of *Cav-1* induced by the activation of tumor promoter 12-O-tetradecanoyl-phorbol-13-acetate (4β-TPA) is associated with enhanced malignancy behavior of endometrial cancer cells through increasing cells anchorage-independent growth, invasion, and migration (Diaz-Valdivia et al., 2015). Additionally, it was reported that *Cav-1* could promote the invasion ability of choriocarcinoma cells, and the repressive expression of *Cav-1* by si-RNA could limit the proliferation and invasion of choriocarcinoma cells (Liu et al., 2008). Perivascular *Cav-1* expression is considered as a marker for distinguishing benign from malignant uterine smooth muscle tumors given that perivascular *Cav-1* is more expressed in leiomyosarcomas compared with uncertain malignant potentials and leiomyomas (Ayaz et al., 2016). The immunoreactivity for *Cav-1* is observed in most cells of leiomyomas. Only few cells in normal myometrial tissues, and mostly co-localized with sex hormone-binding globulin (SHBG) and oxytocin (OT) in leiomyoma samples, which may be linked to growth of leiomyomas (Sendemir et al., 2008).

*The inhibiting role of Cav-1*

Some studies indicate that the normal and elevated expression of *Cav-1* can significantly inhibit tumor cells proliferation, invasion, and metastasis, thus it can be concluded that the function loss and down-regulation of *Cav-1* is a meaningful marker in cancer progression. Sclareol is reported to inhibit cell proliferation and sensitize cells to bortezomib through increasing the expression of *Cav-1* and down-regulating superoxide dismutase1 (SOD1) protein level in cervical cancer cells (Zhang et al., 2017b). Similarly, increased expression of *Cav-1* and mitochondrial carrier homolog 2 (MTCH2) after dihydroartemisinin treatment activate p53, leading to HeLa cell viability inhibition and apoptosis induction (Zhang et al., 2017a). *Cav-1* is critical in the uptake and high cytotoxicity of Cisplatin Nanocapsules through caveolae-mediated endocytosis in ovarian carcinoma cells (Hamelers et al., 2009). It is suggested that *Cav-1* is a critical molecule in the treatment of anti-cancer drug. *Cav-1* can lose its function in cancer repression following inactivation through gene mutation or promoter methylation. *Cav-1* loses its suppressing role in the development of cervical cancer, including 54 SCCs and 18 adenocarcinomas, by methylation of *Cav-1* promoter or other unknown mechanisms (Chan et al., 2003). Having used a new version of Affymetrix Human Genome GeneChip 1.0 ST array, *Cav-1* gene is down-regulated in type I endometrial carcinoma(Saghir et al., 2010). *Cav-1* expression in stromal and perivascular is almost presented in benign ovarian serous tumors, while the *Cav-1* expression is lost in the majority of malignant and borderline tumors, indicating that the expression levels of stroma *Cav-1* can act as a marker to distinguish the benign and borderline ovarian tumors (Sayhan et al., 2015). A previous study indicated that *Cav-1* displayed a tumor-suppressor role, which requiring the presence of *E-cadherin* (Torres et al., 2007). Interestingly, the neo-expression of E-cadherin in previous E-cadherin down-regulated ovarian carcinoma did not inhibit the release and migration of cells from the primary tumor because the expression of *Cav-1* was down-regulated even though E-cadherin level was adequate (Miotti et al., 2005). The simultaneous expression of *E-cadherin* and *Cav-1* is essential to stabilize cell-cell adhesion and play a tumor suppressor role. In addition, hepatocyte growth factor (HGF) down-regulates E-cadherin, beta-catenin, and *caveolin-1*, disassembly of cell-cell contacts, and invasion and migration enhancement in human ovarian cancer cells (Hu et al., 2010). It is suggested that the expression of *Cav-1* can indirectly promote cell-cell adhesion by inhibiting src-related kinases. Thus, down-regulation of *Cav-1* together with increase of src kinase activity might greatly contribute to the migration of ovarian tumor cells by destructing cell adhesion (Prinetti et al., 2011). Another study indicated that *Cav-1* expression was presented at normal ovarian epithelial cells and benign tumor cells, but was absent in serous ovarian carcinoma (Wiechen et al., 2001). Moreover, loss of *Cav-1* can induce disease-associated mutant BRCA1 proteins in serous epithelial ovarian cancer cells (Xu et al., 2014). Overexpression of *Cav-1* can promote apoptosis of ovarian cancer cells after galectin-3 and paclitaxel treatment (Cai et al., 2016). The re-expression of *Cav-1* in OVCAR-3, an ovarian carcinoma cell line, prevents tumor cell survival in vitro. Consistently, *Cav-1* is reduced in ovarian cancer cell lines compared to human ovarian surface epithelial (HOSE) cell lines and up-regulated following progesterone (P4) treatment. Ectopic expression of *Cav-1* leads to growth inhibition, activation of caspase-3, reduction of colony formation, and cell invasiveness in ovarian cancer (Syed et al., 2005). The above results demonstrate that *Cav-1* is down-regulated in human ovarian carcinoma and acts as a candidate of tumor suppressor gene (Wiechen et al., 2001). In uterine leiomyomas, *Cav-1* is down-regulated compared to normal myometrial tissues (Zhou et al., 2014), suggesting that low expression levels of *Cav-1* may promote the pathological process. Therefore, the up-regulation of *Cav-1* expression by gene therapy may significantly inhibit some gynecological tumors progression.

*Perspectives and Conclusion*

*Cav-1* has been reported to play a promoting or inhibiting role in cancer progression in many types of tumors, but there are only a few published studies on the role of *Cav-1* in gynecological tumor. This review summarized some mechanisms of *Cav-1* in cancer progression to profoundly reveal the association between *Cav-1* expression and gynecological tumors. Regardless of *Cav-1* expression in the tumor or stromal cells, we found that *Cav-1* also had paradoxical role in different types of gynecological tumors, both in vivo and in vitro and in the same tumor from the same organ. However, there is no document investigating the intrinsic mechanism of *Cav-1*. Herein, further investigations are needed to study the role of *Cav-1* in the carcinogenesis of gynecological tumors.

**Table 1:** The mi-RNA and Targeting Caveolins in Cancers

mi-RNA	Targeting caveolin	Types of cancer	References
miR-124-3p	*Cav-1*	clear cell renal cell carcinoma	(Butz et al., 2015)
miR-133a	*Cav-1*	head and neck squamous cell carcinoma	(Nohata et al., 2011)
miR107	*Cav-1*	pancreatic ductal adenocarcinoma	(Xiong et al., 2017)
miR-203	*Cav-1*	pancreatic cancer	(Miao et al., 2014)
miR-199a-3p	* Cav-2*	breast cancer	(Shatseva et al., 2011)
miR-218	* Cav-2*	renal cell carcinoma	(Yamasaki et al., 2013)

**Table 2:** The Promoting Role of *Cav-1* in Gynecological Tumor

Types of tumor	The promoting role of *Cav-1* expression	Study type	References
Cervical cancer	Enhancing cell proliferation	*In vivo*	(Sun et al., 2012)
Ovarian cancer	Often expressed in advanced-stage ovarian carcinoma, promoting chemotherapy resistance, inhibiting cell apoptosis	*In vivo* *In vitro*	(Davidson et al., 2001; Zou et al., 2015)
Endometrial cancer	Increasing cells anchorage-independent growth, invasion and migration	*In vivo* *In vitro*	(Diaz-Valdivia et al., 2015)
Choriocarcinoma	Promoting proliferation and invasion	*In vitro*	(Liu et al., 2008)
Uterine leiomyosarcomas	Perivascular *Cav-1* expression in leiomyosarcomas was stronger than that of leiomyomas; *Cav-1* was observed in most cells in leiomyomas and in only few cells in normal smooth muscle cells	*In vivo*	(Sendemir et al., 2008; Ayaz et al., 2016)

**Table 3:** The Inhibiting Role of *Cav-1* in Gynecological Tumor

Types of tumor	The inhibiting role of *Cav-1* expression	Study type	References
Cervical cancer	Inactivation through gene mutation or promoter methylation in cancer repression, inhibiting proliferation and cell viability, and inducing apoptosis	*In vivo * *In vitro*	(Chan et al., 2003; Zhang et al., 2017a; Zhang et al., 2017b)
Endometrial cancer	Down-regulation through Human Genome GeneChip in type 1 endometrial cancer	*In vivo*	(Saghir et al., 2010)
Ovarian tumor	Stromal *Cav-1* regarded as a marker to distinguish benign and borderline tumors, *Cav-1* forming a complex with E-cadherin to stabilize cell-cell adhesion, anti-proliferation, pro-apoptosis and inhibiting invasion, facilitating the uptake of cisplatin nanocapsules	*In vivo* *In vitro*	(Davidson et al., 2001; Wiechen et al., 2001; Miotti et al., 2005; Syed et al., 2005; Hamelers et al., 2009; Sayhan et al., 2015)
Uterine leiomyomas	Down-regulation in human uterine leiomyomas compared to normal myometrial tissues	*In vivo* *In vitro*	(Zhou et al., 2014)
